# Novel genetic advances in schizophrenia: an interview with Michael O’Donovan

**DOI:** 10.1186/s12916-015-0417-1

**Published:** 2015-08-05

**Authors:** Michael O’Donovan

**Affiliations:** MRC Centre for Psychiatric Genetics and Genomics, Hadyn Ellis Building, Maindy Road, Cathays, Cardiff CF24 4HQ Wales

## Abstract

In this podcast, we talk to Professor Michael O’Donovan about the latest genetic advances in schizophrenia based on research data from the Schizophrenia Working Group of the Psychiatric Genomics Consortium. Functional and prediction studies from the identified genetic loci are described together with future directions in psychiatric genetics and its interplay with the environment.

The podcast for this interview is available at http://media.biomedcentral.com/content/movies/supplementary/s12916-015-0417-1-s1.mp3

**Electronic supplementary material:**

The online version of this article (doi:10.1186/s12916-015-0417-1) contains supplementary material, which is available to authorized users.

## Introduction

**Fig. 1:**
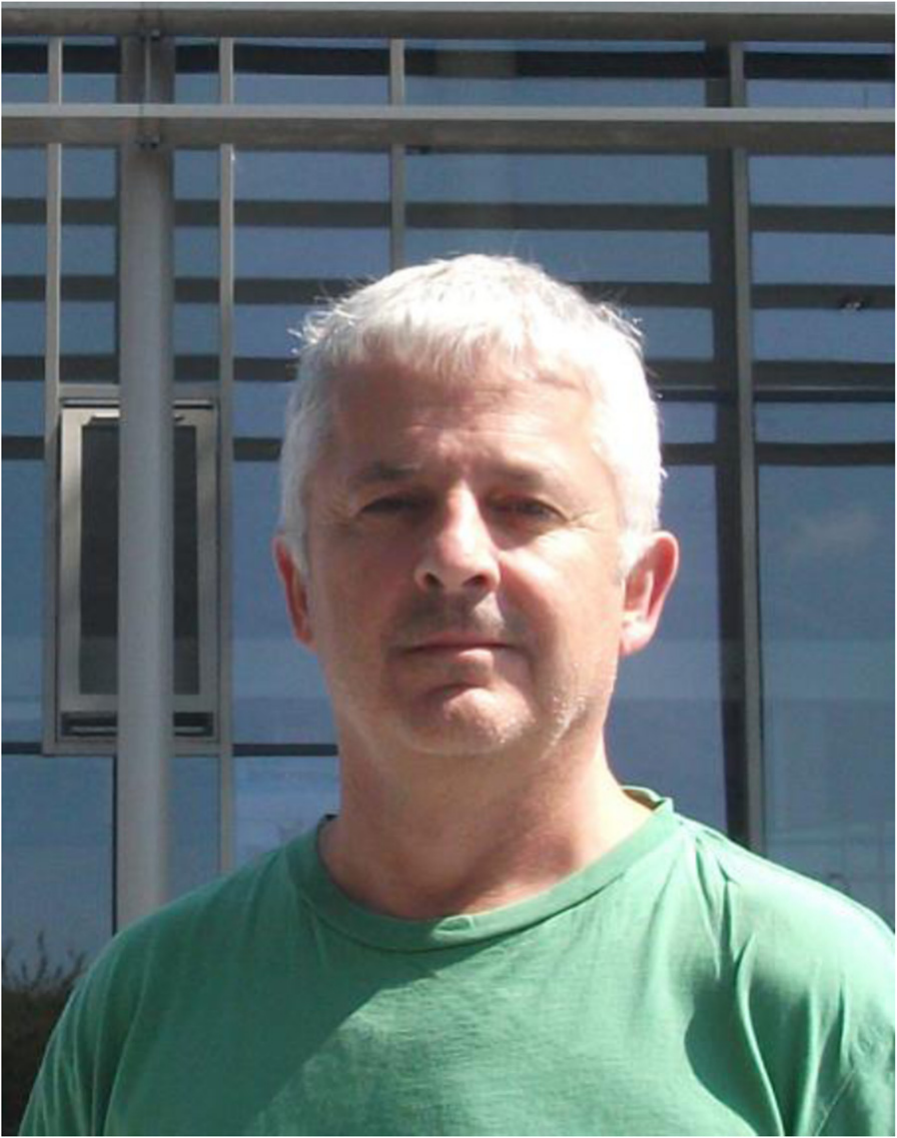
Michael O’Donovan

Professor Michael O’Donovan is a psychiatrist who underwent doctoral scientific training courtesy of the Medical Research Council (MRC) Training and Travelling Fellowship schemes. He works in the Division of Psychological Medicine and Clinical Neurosciences where he is Professor of Psychiatric Genetics and Deputy Director of the MRC Centre for Neuropsychiatric Genetics and Genomics. His clinical work is at the Cardiff and Vale University Health Board. He has a broad interest in the molecular genetics and neurobiology of mental disorders, and, specifically with reference to the contents of the podcast, is the Lead for the Schizophrenia Group of the Psychiatric Genomics Consortium, a group of over 300 researchers from more than 35 countries (Fig. 1).

The podcast for this interview is available at http://media.biomedcentral.com/content/movies/supplementary/s12916-015-0417-1-s1.mp3 (Podcast Q & A: Additional file 1).

## Edited transcript

### 1. You are a leading expert in the molecular genetics of major psychiatric disorders including schizophrenia. Can you explain why you decided to specialise in this particular area?

During my medical training, I found psychiatry to be an interesting subject, which is why after completing medical school I went on to train as a psychiatrist. During that time, I became very interested in schizophrenia. It was clear that people did not know a lot about what caused schizophrenia, but that genetics was a pretty promising clue. That, together with some inspiration from Professor Peter McGuffin, who was Head of Department in Cardiff when I was a Lecturer, and later Professor Mike Owen, got me into genetics. Since my early training in the subject, the techniques of molecular genetics constantly evolved until finally, after a very long slog, it has been possible to actually use the principles of genetics to make some discoveries in this complicated field. So, the usual mixture of interest, luck, and being in the right place at the right time is what got me into the genetics of psychiatric disorders in general and schizophrenia in particular.

### 2. This has resulted in several major findings in the field. Recently, you have been the lead author on the largest molecular genetics study of schizophrenia that was published in *Nature* last year [1]. This study came about as a result of the Schizophrenia Working Group of the Psychiatric Genomics Consortium. Can you describe why this consortium was formed and tell us what genotype data it included?

Over the last 20 years, researchers have been working initially on their own samples, which were often a hundred cases and a hundred controls, or so. By 2001, even the larger samples increased to only about 500 cases and 500 controls. At the same time, it became increasingly clear from developments that were taking place in the wider world of genetics, for example in the field of diabetes, that the effect sizes of risk alleles we were looking for, or the amount of population risk that any one genetic variation contributed to, was likely to be small. As a result, people began to recognise that we needed sample sizes well above those that individual groups were studying. So in the mid-2000s, consortia were formed.

I was part of the International Schizophrenia Consortium, and even then the sample sizes were a few thousand cases and a few thousand controls. After the Wellcome Trust Case Control Consortium Study of Seven Common Diseases, it was pretty clear that even these samples were not powerful enough to make much of a dent in the genetics of the disorder, and that turned out to be true when those consortia analysed their results. So, the consortia themselves started to amalgamate; people in these smaller consortia began to reach out to everyone they knew who was working in the field to try and bring the samples together, with the hope of getting a decent-sized sample with power to make clear and unambiguous findings. Pat Sullivan was a major driving force here in psychiatry in general, and within schizophrenia, Pablo Gejman led the first Psychiatric Genomics Consortium (PGC) study. It has really been, once again, a process of evolution; people have recognised that the sample size was a problem, and they have banded together in increasingly large numbers to contribute their samples and their genetic data.

For this particular study, the vast majority of the investigators have made what we call the ’individual genotypes’ available to the consortium. Each group mostly performed their own genome-wide association studies (GWASs) and they contributed the individual level genotypes into a central data repository in the Netherlands. This included groups from 34 different countries across the globe. As well as Europe and North America, it also included a number of groups from Australia and from Asia, and in particular Japan, Singapore, and some groups with Chinese samples. It was truly an international collaboration - and it’s getting bigger.

We undertook a meta-analysis of all of these raw individual genotype data, and then at the end we sought replication from a separate consortium that for legal reasons could not contribute all of its data. That consortium was led by Decode Genetics in Iceland, and they contributed summary statistics in a format that allows us to combine their results with our primary GWAS results.

### 3. The Schizophrenia Working Group of the consortium really enabled a large number of cases and controls in the meta-analysis. Could you briefly describe the findings of the study?

I expect that people may be aware that in these surveys of essentially the whole of the genome for common genetic variations, we require very stringent statistical thresholds to recognise a finding as being very likely a true positive rather than a statistical chance aberration. We found over 120 such associations that surpassed this threshold, which means that there is a very, very strong chance that these things are true. When we distilled that down and accounted for multiple associations to the same genomic region, we found that we had 108 independent findings. So, there were 108 physically distinct chromosome regions associated with schizophrenia. Each of those regions, in turn, contains one or often more than one gene, so the next task is to try and discover how association points to change in gene function.

To explain the actual findings, first of all I would like to make an important caveat for those people who are not geneticists: an association does not pinpoint a gene. An association points to a region of a chromosome. As I said a moment ago, some associated regions contain multiple genes that could be driving this association signal. Some contain apparently only one gene that could be driving the signal. However, there are as yet unrecognised functional elements in the genome. Thus, it is actually possible, for example, that if we find an association to a region that contains only one gene, it might be a functional element that drives changes in a gene some distance away that we are not yet aware of. However, in general it seems to be a valid assumption that the relevant genes that are driving associations are those that are very close to that genetic variant that shows association.

With that important caveat in mind, what we actually found was that, as is generally the case in studies like this in other diseases, the associations were to regions containing a whole host of genes that one would never particularly suspect to be involved in schizophrenia. For many of these genes, we do not even have the faintest clue about their function.

We also found a number of associations that appear to point to historical genetic favourites. Schizophrenia, being a somewhat enigmatic disorder in terms of its underlying biology, has been linked to many hypotheses about how the disorder is caused. Some of the leading hypotheses concern alteration in dopamine function, and particularly a receptor called dopamine D_2_ receptor (DRD2), which is the target of all antipsychotic drugs. We found an association that would probably implicate that gene as being involved in the basic aetiology of schizophrenia. We additionally found associations in and around a rather large number of genes that affect glutamate function, which is also one of the more important longstanding neurochemical hypotheses of schizophrenia.

Thus, we found a range of findings that pointed to some old hypotheses being at least in part relevant to the pathophysiology of schizophrenia. However, most of the findings point to the possibility of novel genes. Really the work is just beginning to try and figure out how those associations may relate to the pathophysiology of the disorder.

### 4. To explore the regulatory nature of the identified genetic variants, they were tested in different cell lines and tissues. What were the results of these functional investigations?

One way of trying to pursue how genetic associations might relate to broad areas of biology is to do what are called gene set analyses, pathway analyses, or network analyses, where one takes all the findings and tries to link them to groupings of genes that are perhaps expressed in certain tissues or are involved in certain biological processes.

One of the approaches that we took was to use publicly available Encyclopaedia of DNA Elements (ENCODE) data. ENCODE is studying the regulatory elements of the human genome. What we did was to extract from that data DNA regulatory elements that appear to be most active in particular tissues and cells, and test whether the genetic associations are enriched in and around those regulatory elements. For example, is there an excess of associations around regulatory elements that appear to be active in neurones or in lymphocytes or in the gut or in the pancreas?

In general, we found two main things using that approach. We found that gene regulatory elements that appear to be active in brain tissue and neuronal cell lines were particularly enriched for schizophrenia associations, suggesting, as would have widely been expected, that schizophrenia is in a substantial way a disorder of the brain rather than a disorder of the gut or gut absorption or many of the other hypotheses that been voiced in the history of schizophrenia but still persist.

One of the more intriguing novel findings was the enrichment of association in regulatory elements that are particularly active in a number of immune tissues. Again, all this type of work always comes with an important caveat. When we see enrichment of association signals in the vicinity of regulatory elements that appear to be active in immune tissues, what we are not yet sure of is whether those regulatory elements are specific for immune tissues. Could it be that those regulatory elements are also particularly active in the sorts of tissues that you would expect like the brain?

We did try to statistically allow for that, and it would appear that there was an independent enrichment of the association signal. Regulatory elements that we did not find to be particularly active in the brain but were active in these immune tissues were also enriched for signals. However, there are a number of unanswered questions at the moment including which genes are exactly affected in the immune tissues. Do these genes only have immune functions or do they have functions in the brain? What change in function is indicated by an association? This is why, in our manuscript, we posed some caution about the interpretation of the immune finding. In some ways this was one of the more interesting results because, if it is true, it supports very longstanding hypotheses that schizophrenia involves sub-optimal immune responses, for example, excessive responses to infection or perhaps some forms of autoimmunity. However, one does not want to jump too far in coming to that conclusion based purely upon genetic data.

### 5. Another finding was of overlap of the novel genetic loci with *de novo* genetic mutations. Can you briefly describe what that analysis showed?

This analysis was perhaps quite an unexpected finding in many quarters. The background to this is that, in parallel to these common genetic variant studies that have been taking place in schizophrenia, we and others have also been studying rare genetic variation in the disorder in the form of copy number variation, but more recently through DNA sequencing looking for point mutations.

We have studied both *de novo* copy number variants in schizophrenia and *de novo* point variations in schizophrenia. It is now clear that these new mutations - ones that occur in a person but not in either of his or her parents - contribute to the disorder in some people with schizophrenia. It is not a high proportion, but these may be important mutations because in theory their effect sizes might be much bigger than these common genetic variants that we have been discussing which individually have very small effect sizes.

Having identified genes with these very rare new mutations in our earlier work, we now looked to see if these cluster more than one would expect in the same regions that are showing genome-wide association. We found evidence that they do. Moreover, we also showed that genes that show similar types of *de novo* mutations for other neurodevelopmental disorders also tend to cluster in the schizophrenia associated regions. This supports the broad hypothesis that many of the neurodevelopmental disorders are, to a certain extent, aetiologically linked. Here I am referring specifically to intellectual disability and to autism spectrum disorder, though the evidence is wider and includes for example ADHD as well. This is further evidence that there is some kind of commonality between these disorders. I think of them as sharing part of their aetiology, not all, and for the bit in common, they differ to a certain extent by the severity of the mutation type.

The other important implication of that finding of overlap is that one of the criticisms that people levy at researchers who are studying rare genetic variations is: “This is some kind of weird subtype of the disorder that does not really have any relevance to the disorder as a whole in the general population.” The modest degree of convergence between rare genetic variation and common genetic variation suggests that the findings that one might make from these kinds of rarer, bigger effect mutations will have biological relevance for the general population of people with schizophrenia. The reason why this is important is that, in many respects, when one is doing follow-up functional studies (modelling them in cell lines or in animals) it is easier to do modelling with the rare genetic variation that tends to have larger effects on the disorder, compared with those with rather weak genetic effects.

### 6. An additional part of the study determined the risk of the profile scores to predict the presence or absence of the disorder. Can you briefly explain how the genetic data obtained could predict case control status?

Although we have detected quite a large number of associations, they are the tip of the iceberg. Say we don’t just take these very stringently associated genetic variants but rather go down the list to more weakly associated genetic variants, those that we call ’sub-threshold’ associated. These met some level of significance, but as we go further down the list of significance, we become less confident that any given individual allele is associated with the disease. Nevertheless, the theory is that those that show some evidence for association should be relatively enriched for true associations, even though as we go down the list, for any true association, we drag in many more false ones.

In this way, we consider all alleles that are more significant than some nominal threshold as ’potential risk’ alleles. We derive them from one dataset; let’s say there are 10,000 of these alleles that surpass some kind of statistical threshold. Subsequently, in an independent sample, and simplifying this procedure to some extent, one simply counts up how many of these risk alleles each person carries. The prediction is that cases from a different study will have a higher number of these risk alleles than the controls do. What we call the risk profile score is a weighted average of the number of these risk alleles carried. That this approach could statistically differentiate between cases and controls was shown by a study that was published in *Nature* by the International Schizophrenia Consortium. However, in the early days the predictive power enabled statistical differentiation between cases and controls, but the sensitivity and specificity were extremely poor. However, as the genome-wide association studies became larger, the power to achieve this discrimination has improved.

So the principle works certainly, but it is important to note in practice that we still cannot use this for a clinical diagnosis. Although we have high statistical significance - we can obtain very, very small *P*-values concerning group differences between cases and controls - when it comes to looking at an individual person, although cases have on average a higher score than the controls, many of the controls have scores higher than many of the cases. In other words, the score distribution from cases overlaps substantially with the score distribution from controls, and as an upshot, we cannot confidently predict who is a case and who is a control based only on their polygenic score.

What we can do in a research setting is screen large population samples which have GWAS data and find people who are at relatively higher risk for schizophrenia, even though the vast majority of them will develop schizophrenia, and at the other end of the score distribution, people who are at relatively low genetic risk for the disorder, even though a small proportion of them will in fact develop the disorder. We can then start doing interesting studies about how those two populations differ in terms of biology to try to find out what it is about brain function that relates to high and low risk. The profile scores are useful in a research setting, but they are of no current value in a clinical setting.

### 7. Can you describe the future directions in the field of psychiatric genetics and how it will be influenced by the interplay with the environment?

In principle, the simplest thing that we and others are trying to do is increase the sample sizes, because although we have made many discoveries so far, there is much more to be done. The aim of the Psychiatrics Genomics Consortium is, over the next four years perhaps, to try and get GWAS data on 100,000 cases instead of the current number in the mid-30,000s. Thus, we are looking for more power and more findings.

It is also important to try and achieve much better functional interpretation of the common genetic findings. That will require better functional annotation of the genome, in terms of which genetic variants regulate genes in which cell types and in which areas of the brain at what developmental times including foetal life. A lot of background biology is required to try and provide resources that allow us to interpret common genetic findings.

We and others are also interested in trying to use the common genetic findings and rare genetic results to sub-classify the heterogeneous disorder into more homogeneous groups. So far, attempts to carve it up by symptom pattern or age at onset, or in various other ways that one might conceptualise, have not really produced convincing results. The reason why we are trying to get more homogeneous populations of people with the disorder is that such membership of distinct groups might be clinically useful, for example, in trying to figure out why some treatments work in some people but not in others, and in overall improving the treatment prospects.

There is also a lot of work going on in rare genetic variation. Increasingly, people are adopting sequencing approaches - initially exome sequencing as we have done looking for *de novo* mutations, but over the next few years we expect there to be larger samples that will be analysed by whole genome sequencing. Here the objective is that rare genetic variation contributes to some genetic risk, although most of us do not believe that rare genetic variation contributes to the majority of the genetic risk. As I mentioned earlier on in this podcast, it is easier to model the mutations in cells and animals and thereby probe what kind of biological consequences genetic variants have on cellular function and whole organism function, respectively.

Researchers are also interested in taking the genetic findings and figuring out what kind of physiological changes occur in people without the disorder. Perhaps this can be indexed by observing changes in brain function using functional imaging techniques or varying other methods for assaying brain function. Additionally cognitive function can be determined in terms of measuring brain performance. We in Cardiff, along with colleagues in London led by Shitij Kapur, are trying to use various genetic profiling techniques to try and predict who will and who will not respond to particular treatment types. We regard the genetic findings really as an infrastructure for a whole range of future investigations. It is impossible for me to predict or detail every single way that this is likely to be exploited; the options are almost boundless.

There is certainly an environmental contribution to schizophrenia. We have some clues that perhaps perinatal infections or other forms of adverse challenges during pregnancy and surrounding childbirth contribute. There is evidence that very traumatic experiences in childhood may contribute, and that in certain populations that migration, possibly as a result of stress or discrimination, contributes to the disorder. Thus, there are a range of clues about the environment. What is more controversial is whether or not environmental exposures in general increase your risk or only increase your risk with a given genetic background. The latter is often referred to as ’gene-environment interaction’.

Environmental studies that include genetic data, and genetic studies that include environmental data, are sub-optimal at the moment. The power to detect so-called gene-environment interactions is significantly less than it is to identify the genetic effects in the first place, although studies are proceeding to try and see how particular genes or whole genetic risk relates to drug abuse in terms of dictating schizophrenia risk. In fact, I and my colleagues, led by Jim van Os at the University of Maastricht, have such a study funded by the European Union. I expect that the main deliverables from that will take several more years, because I suspect the sample sizes are currently inadequate.

### 8. Where can I find out more?

See the reference list [1–10].

## Additional file

Additional file 1:
**Q & A an interview with Michael O’Donovan.**
